# COVID-19 Inequalities: Individual and Area Socioeconomic Factors (Aragón, Spain)

**DOI:** 10.3390/ijerph18126607

**Published:** 2021-06-19

**Authors:** Isabel Aguilar-Palacio, Lina Maldonado, Sara Malo, Raquel Sánchez-Recio, Iván Marcos-Campos, Rosa Magallón-Botaya, Mª José Rabanaque

**Affiliations:** 1 Preventive Medicine and Public Health Department, University of Zaragoza, 50009 Zaragoza, Spain; smalo@unizar.es (S.M.); rsanchez@unizar.es (R.S.-R.); rabanake@unizar.es (M.J.R.); 2 Instituto de Investigación Sanitaria de Aragón, IIS Aragón, 50009 Zaragoza, Spain; lmguaje@unizar.es (L.M.); imarcos@iisaragon.es (I.M.-C.); rosamaga@unizar.es (R.M.-B.); 3 Grupo de Investigación en Servicios Sanitarios de Aragón (GRISSA), IIS Aragón, 50009 Zaragoza, Spain; 4 Department of Economic Structure, Economic History and Public Economics, University of Zaragoza, 50009 Zaragoza, Spain; 5 Department of Medicine, Psychiatry and Dermatology, University of Zaragoza, 50009 Zaragoza, Spain

**Keywords:** COVID-19, coronavirus infection, inequalities, socioeconomic factors

## Abstract

It is essential to understand the impact of social inequalities on the risk of COVID-19 infection in order to mitigate the social consequences of the pandemic. With this aim, the objective of our study was to analyze the effect of socioeconomic inequalities, both at the individual and area of residence levels, on the probability of COVID-19 confirmed infection, and its variations across three pandemic waves. We conducted a retrospective cohort study and included data from all individuals tested for COVID-19 during the three waves of the pandemic, from March to December 2020 (357,989 individuals) in Aragón (Spain). We studied the effect of inequalities on the risk of having a COVID-19 confirmed diagnosis after being tested using multilevel analyses with two levels of aggregation: individuals and basic healthcare area of residence (deprivation level and type of zone). Inequalities in the risk of COVID-19 confirmed infection were observed at both the individual and area level. There was a predominance of low-paid employees living in deprived areas. Workers with low salaries, unemployed and people on minimum integration income or who no longer receive the unemployment allowance, had a higher probability of COVID-19 infection than workers with salaries ≥ €18,000 per year. Inequalities were greater in women and in the second wave. The deprivation level of areas of residence influenced the risk of COVID-19 infection, especially in the second wave. It is necessary to develop individual and area coordinated measures by areas in the control, diagnosis and treatment of the epidemic, in order to avoid an increase in the already existing inequalities.

## 1. Introduction

The 2019 coronavirus disease (COVID-19) outbreak in China has triggered an unprecedented global public health crisis [1]. On 11 March 2020 the World Health Organization (WHO) declared this outbreak a global pandemic [2]. According to WHO COVID-19 Dashboard [3], by March 2021 there were more than 120 million confirmed cases and more than 2.6 million deaths worldwide. Spain has been one of the European countries most affected by the COVID-19 pandemic. At the time of writing this article, Spain has more than three million confirmed cases and a 14-day incidence rate of more than 140 cases per 100,000 inhabitants, with a lethality of 2.3% [4].

As described in previous public health crises, pandemics do not affect people uniformly [5]. Inequality patterns are observed both at the global and local scales, showing worse health outcomes in populations and areas with lower socioeconomic levels [6,7]. Numerous studies have shown that the most disadvantaged social classes, such as poor, certain immigrant groups, black people and indigenous people, are more vulnerable to infectious diseases than more advantaged social groups [8,9,10]. This vulnerability has been associated with social overcrowding, lack of health literacy and lack of access to vaccinations, health services, food and basic hygienic measures, among others.

Regarding individual socioeconomic characteristics, several authors have pointed out a socioeconomic gradient in COVID-19 outbreaks due to differences in knowledge and practices towards COVID-19 [11,12]. In this sense, individual socioeconomic position has a direct effect on the risk of infection and the appearance of severe disease consequences [13]. In relation to the type of job, low-paid workers have a higher probability of being designated as key workers, with the consequent increased risk of exposure [14]. Other individual factors that can explain these differences have also been described, such as living below the poverty line, lack of health insurance, lower health literacy, higher medical susceptibility or higher exposure rates, associated with household crowding or multigenerational living [15,16].

However, socioeconomic differences do not only play a fundamental role at the individual level. Other levels of aggregation, such as area of residence, are key to understand the existence of inequalities. In Spain, COVID-19 studies conducted in the area of residence [17,18] showed that COVID-19 incidence was higher in the most deprived urban areas. In this sense, it has been described that living in disadvantaged environments is related with the existence of chronic stressors that, after a time, damage the health of its inhabitants [14]. Living in a deprived area is also associated with poorer access to health care, even in universal healthcare systems [19], dependence on public transport or living in small places shared with other people, where the adoption of appropriate quarantine measures is not possible [20].

Significant variations have been observed in the evolution of the pandemic in Spain. Administratively, Spain is organized into 17 Autonomous Communities with independent healthcare management. Aragón is a northeastern Autonomous Community of 1.3 million inhabitants. COVID-19 pandemic has had a strong impact on this population, with more than 110,000 confirmed cases at the moment of writing this article [21]. Moreover, Aragón has shown certain differences in the COVID-19 pandemic with respect to the rest of the Autonomous Communities in Spain. The main difference is that, unlike the rest of Spanish Autonomous Communities, with three waves, Aragon has registered four waves at the time of writing this article, with varying social and healthcare impacts, and changes in the profile of affected individuals [21]. This is explained by the fact that the second wave in Aragon started earlier than in the rest of the country, being related to the arrival of seasonal fruit pickers to certain farming areas, as well as to the presence of urban neighborhoods where the population of the most disadvantaged social class and related to these seasonal workers is concentrated. The analysis of these variations is crucial to understand the evolution of COVID-19 spread and the effect of the measures adopted.

In this context, inequalities in the risk of COVID-19 infection are to be expected, with the most vulnerable groups having a higher risk of infection. Therefore, understanding the impact of social inequalities on the risk of COVID-19 infection is essential when designing strategies to reduce COVID-19 incidence, in order to mitigate the social consequences of the pandemic. To this end, the objective of our study is to analyze the effect of socioeconomic inequalities, both at the individual and area of residence levels, on the risk of COVID-19 confirmed infection in Aragón (Spain) and its variations throughout the three waves of the pandemic.

## 2. Materials and Methods

### 2.1. Design, Information Sources and Study Population

We conducted a retrospective cohort study using data from the Aragón-COVID19 cohort. This is a health data collection of all individuals undergoing COVID-19 testing in the Spanish region of Aragón. Aragon is an Autonomous Community located in the northeast of Spain. It is the fourth Spanish Community by extension but occupies the 11th place of 17 in terms of population. It has a population of 1.3 million inhabitants and half of the population live in the city of Zaragoza. Their level of aging is high, with 21.7% of people over 64 years of age [22]. Regarding socioeconomic level, it has an average level within the country with some indicators, such as the unemployment rate, showing a better situation than the national average [22]. Public health care, which covers practically the entire population, is structured in 8 health sectors organized into 123 Basic Healthcare Areas (BHA), each of them served by a primary care center and with populations between 2000 and 5000 inhabitants [23].

People included in the Aragón-COVID19 cohort were tested either when they had symptoms compatible with COVID-19 or when they had close contact with a confirmed subject. The Aragón-COVID19 cohort includes information gathered from administrative health data sources as well as electronic health records of the Aragón health service. All individuals in the cohort were included from 9 March 2020, the first epidemiological week with COVID-19 cases reported in Aragón, to 13 December 2020, the latest data available at the moment of writing this paper (357,989 individuals). All COVID-19 cases were confirmed using polymerase chain reaction (PCR) or COVID antigen testing. 

The research protocol of this study was approved by The Clinical Research Ethics Committee of Aragón (CEICA) (PI20/184).

### 2.2. Variables of the Study

We analyzed the sociodemographic and clinical information of all the individuals in the cohort. Regarding sociodemographic characteristics, we consider sex, age (under 15, 15–44, 45–64, 65–79 and 80 years or older), and socioeconomic level. Socioeconomic level was calculated on the basis of pharmacy copayment levels and social security benefits received, according to the type of user of the Aragón health service. From the combination of these two variables, 8 mutually exclusive categories were obtained: employed individuals earning less than €18,000 per year, employed individuals earning €18,000 per year or more, individuals receiving the unemployment allowance, individuals with a contributory pension of less than €18,000 per year, individuals with a contributory pension of €18,000 per year or more, individuals affiliated to the mutual insurance system for civil servants, individuals receiving free medicines (people with minimum integration income or who no longer receive the unemployment allowance), and other situations not previously considered. The clinical information included was obtained from the morbidity adjusted groups (GMA) [24]. This source of information considers all medical diagnoses available in primary healthcare and hospital discharge records (CMBD). We considered GMA information from January 2020 in order to know the health status prior to the COVID-19 diagnosis of the cohort individuals. The three variables analyzed from GMA were weight complexity (obtained from the aggregation of the patient’s different diagnoses), the presence of chronic morbidities and the presence of respiratory illnesses.

We also considered two additional variables by BHA of residence. The first variable was the BHA deprivation index categorized into four quartiles, from least (Q1) to most (Q4) deprived. This deprivation index combines information of four indicators from the Population and Housing Census 2011 (last available): percentage of unemployment, percentage of temporary workers, percentage of people between 16 and 64 years with low educational level and percentage of immigrants [25]. The other variable obtained by BHA was the classification of the zone into rural or urban, according to the Aragon Government [26]. Accordingly, urban areas are those that concentrate at least 80% of the BHA population in their municipalities and rural areas are those that do not meet this criterion. 

The summary of the variables, their description and categories can be consulted in Table A1.

### 2.3. Model Specification

Analyses were performed both globally and considering the three existing pandemic waves in Aragon until December 2020: from March 9 to June 21; from June 22 to October 11; and from October 12 to December 13. All analyses were stratified by sex.

We described sociodemographic and clinical characteristics of all individuals included in the cohort, globally and according to COVID-19 confirmed diagnosis. Sociodemographic and morbidity differences by wave in individuals with a laboratory-confirmed COVID-19 infection were described. Categorical variables were described by percentages. Weight complexity had a non-normal distribution, so median and interquartile range were used to describe this variable. Statistical differences between waves were assessed using chi-square and Mann–Whitney tests.

In order to study the effect of inequalities on the risk of having a diagnosis of COVID-19, multilevel analyses stratified by sex were developed (1). Analyses were conducted for the entire period analyzed and by pandemic wave. Two levels of aggregation were considered: individuals and BHA. Each individual included in the study has his/her own characteristics in terms of age, socioeconomic status and previous morbidities, but they also belong to a particular BHA, each with different characteristics in terms of deprivation index and type of BHA (rural or urban). When data are grouped together, there is an intra-class correlation, meaning that there are observations that are more similar to others in the same group than to those in other groups. When adjusting the multilevel model using random intercepts, part of the variability in the response variable is divided into each “level” (deprivation index and BHA type, respectively) and variance partition coefficients can be calculated to see how much of the variance of the response belongs to each level.

Individuals could simultaneously belong to more than one group of a given hierarchical level. Thus, at the same time, an individual belongs to a BHA with a given deprivation index and to a rural or urban BHA. This leads to a cross-classified structure. In this case, we classified COVID cases by their BHA deprivation index (quartiles) and type of zone (urban or rural), so that both are considered to be random. Cross-random effects are used when each category of one factor co-exists with each category of the other factor (there is at least one observation of categories for both factors).

(1) $$COVID_{i\left(sj\right)}=log\frac{\pi_{sj}}{1-\pi_{sj}}=\beta_{0}+{\left(X\beta \right)}_{i\left(sj\right)}+u_{s}+u_{j}+e_{i\left(sj\right)}$$

with $\pi_{sj}=P\left(y_{sj}=1\right)$, when an individual i belongs to a rural or urban BHA *s* (*s* = 1 (*rural*), 2 (*urban*)) and deprivation index *j* (*j* = 1,..4 -*Quartiles*-).

In this model, X set of explanatory variables includes K regressors. Individual sociodemographic characteristics (age and socioeconomic level) and morbidity were considered as explanatory variables. The parameter β represents the fixed effects. This model has three assumptions: first, the random effects $u_{s}$ y $u_{j}$ are normally distributed with mean 0 and variance $\sigma_{u}^{2}=\sigma_{\beta }^{2}$, which represents the differences in the self-referred hospitalization use variable attributable to the country; second, the error component $e_{i\left(sj\right)}$ is also normally distributed with mean 0 and variance; third, the random effects $u_{s}$ y $u_{j}$ and the error component $e_{i\left(sj\right)}$ are independent, and $e_{i\left(sj\right)}$ are all independent of each other. Interactions between variables were systematically investigated and collinearity was considered. Finally, the likelihood ratio test (LR test) was used to evaluate the final model. The significance of the fixed effects was also evaluated with the Wald Test.

All analyses were performed using R Statistical Software (the R Foundation for Statistical Computing, Vienna, Austria). Data were analyzed using a linear mixed-effects regression based on the lme4 package [27] in R statistical package version 4.0.4.

## 3. Results

### 3.1. Aragón-COVID19 Cohort Description

Data from 357,989 individuals included in the Aragón-COVID19 cohort were analyzed. Of these individuals, 74,039 (20.7%) had a COVID-19 confirmed infection. 53.4% of the studied population were women, with a COVID-19 positivity of 20.5%. In the case of men, positivity was 20.9%. Positivity rates were similar between men and women. In women, the age groups with the lowest and highest positivity rates were, respectively, the youngest and the eldest group. In men, the lowest positivity rate was observed in those < 15 years old, while the highest positivity rate was found in people from 45 to 64 years old (23.84%). Regarding socioeconomic status, those with free medicines in women (22.22%) and with “other” category in men (22.33%) showed the highest positivity rates. For both sexes, positivity rates were slightly higher in the most deprived quartile and similar in the rural and urban context. In terms of clinical characteristics, the highest positivity rates were observed in those people with a hospitalization.

Sociodemographic and morbidity descriptions of all individuals studied are available in Table A2 and Table A3. There were statistical differences between people with no COVID-19 diagnosis and COVID-19 confirmed cases for age, socioeconomic level, deprivation quartile and hospitalization for both men and women. In the case of women, those without a confirmed COVID-19 diagnosis also presented a higher prevalence of respiratory illnesses. In men, differences were observed for all clinical variables considered. 

### 3.2. Sociodemographic and Morbidity across Waves

When comparing sociodemographic and morbidity profiles of confirmed COVID-19 cases across waves, we observed significant statistical differences for all variables evaluated. In women, the age group with more COVID-19 confirmed cases was 15 to 44 years old for waves 2 and 3 (Table 1). Regarding individual socioeconomic level, the highest frequency of confirmed cases in wave 1 was observed in pensioners with low income (34.4%), while workers with low salaries showed the highest frequency of COVID-19 confirmed cases in waves 2 and 3. When deprivation was analyzed by BHA of residence, 32.9% of confirmed cases lived in the least deprived areas in wave 1. This changed in wave 2, with the highest percentage of COVID-19 cases living in the BHA with the highest deprivation. The highest frequency of confirmed cases in urban areas was observed for wave 2. Weight complexity, presence of chronic morbidities and respiratory illnesses were significantly higher (*p* < 0.001) in wave 1 than in the other two waves. 

We observed similar results in men as in women (Table 2) for individual socioeconomic level, BHA deprivation, type of BHA (rural or urban) and previous morbidities. 

### 3.3. Inequalities in the Risk of Having a Diagnosis of COVID-19

The results of the multilevel analysis in women (Table 3) showed a high risk of COVID-19 infection with increasing age, with the highest risk being observed in the elderly (odds ratio (OR) 2.5; 95% confidence interval (95%CI): 2.3–2.7 for the global model). We observed socioeconomic inequalities in the risk of COVID-19 infection, especially in wave 2. Thus, those women with free medicines (women with minimum integration income or who no longer receive the unemployment allowance), workers with low salaries and unemployed presented a higher risk of COVID-19 infection than those workers with salaries ≥€18,000 per year. Finally, women with previous chronic morbidities showed a lower risk of COVID-19 infection than those with no morbidities, after adjusting for the rest of the variables of the model (OR: 0.8; 95%CI 0.8–0.9 for the whole period analyzed).

The highest value of the between-group variance (τ00) was observed in phase 2 for the deprivation quartile (0.0209). This result shows that about 2.09% of the residual variance of the dependent variable (COVID infection) is attributable to differences between deprivation quartiles, after controlling for the explanatory variables. There were differences in the risk of COVID-19 infection depending on BHA of residence, and especially by deprivation quartile. This effect was greatest in wave 2, with a median OR of 1.15 for BHA deprivation and 1.11 for zone of residence. In all cases, the models with varying intercepts among crossed random effects fit the data significantly better than other models.

In both men (Table 4) and women, there was a high probability of COVID-19 infection with increasing age. The highest risk of COVID-19 infection was observed in the elderly in wave 1 (OR: 10.9; 95%CI 7.4–15.9) in relation to the youngest group (<15 years old). In men, socioeconomic inequalities in the risk of COVID-19 infection were also observed, especially in wave 2, but these differences by socioeconomic level were lower than in women. Thus, in wave 2, workers with low salaries had a higher risk of COVID-19 infection than those workers with higher salaries (OR: 1.2; 95%CI 1.1–1.2). This result was also observed in men with free medicines (OR: 1.2; 95%CI 1.1–1.3). Regarding the existence of previous morbidities, a lower probability of COVID-19 infection was observed in those with chronic morbidities than those with no morbidities, after adjusting for the rest of the variables in the model (OR: 0.9; 95%CI 0.8–0.9 for the entire period). As observed in women, the highest value of τ00 was observed in wave 2 for the deprivation quartile (0.0255), whereas the highest value of τ00 for zone of residence was obtained in wave 1 (0.0071). Median odds ratio showed its largest effect in wave 2, with similar values to those in women for both, deprivation quartile and zone of residence.

**Figure 1:**
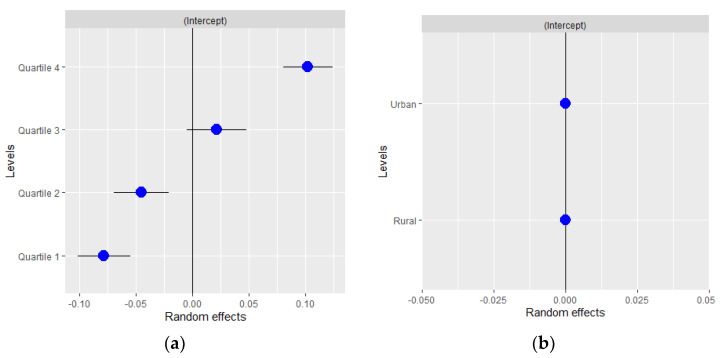
Random effects by (**a**) basic healthcare area deprivation and (**b**) by type of area in women for all the period analyzed.

**Figure 2:**
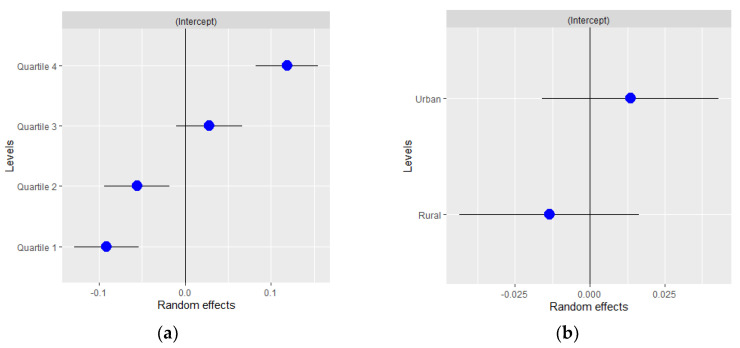
Random effects by (**a**) basic healthcare area deprivation and (**b**) by type of area in men for all the period analyzed.

Finally, Figure 1 and Figure 2 show the results of the random effects and their standard errors for each of the variables at the area level for the entire period analyzed. As can be observed, in women (Figure 1), differences in random effects were found between deprivation quartiles. Thus, the risk of COVID-19 infection was lower in the least deprived quartiles (quartiles 1 and 2) and significantly higher in the most deprived (quartile 4). This effect was also observed in men (Figure 2). In contrast, no differences in random effects were found in relation to the type of area.

## 4. Discussion

### 4.1. Main Results

The objective of this study was to explore the existence of individual and area inequalities in the risk of COVID-19 confirmed infection, and its variations across three pandemic waves (from March to December 2020). As we have observed, Aragón has been severely affected by the COVID-19 pandemic, with high incidence rates in all age groups, especially in the young active population and the elderly. COVID-19 incidence rates were higher in women than in men. Different profiles of patients with confirmed COVID-19 diagnosis have been observed among the total who were tested in the three waves analyzed, with the most striking changes between wave 1 and waves 2 and 3. In wave 1, the highest frequency of confirmed cases was observed in low-income pensioners, with a high prevalence of chronic morbidities and living in BHA with low deprivation index. On the contrary, in waves 2 and 3 there was a predominance of employees with low salaries and people living in deprived BHA. This profile was similar for both sexes. Regarding multilevel analyses, there were inequalities in the risk of COVID-19 infection according to individual socioeconomic status. Taking workers with salaries ≥€18,000 per year as reference, workers with lower salaries, the unemployed and people with minimum integration income or who no longer receive the unemployment allowance, had a higher risk of COVID-19 infection. These inequalities were greater in women and in wave 2. The deprivation level of BHA of residence influenced the risk of COVID-19 infection, especially in wave 2. 

### 4.2. Differences Across Waves

When analyzing the evolution of the pandemic in Aragón, the large difference in incidence rates between the first wave and the other two is striking [21], which is probably related with the test availability and the lack of clear diagnosis protocols at the beginning of the pandemic. As stated by Marí et al. [18], the first Spanish wave was based on hospitalized cases. This is the reason why it affected mainly the elderly and people with chronic conditions. This fact would also explain the differences in wave 1 versus wave 2 and 3 in terms of inequalities, as testing accessibility improved during the pandemic, revealing inequalities that had been hidden at the beginning [28]. Finally, the low risk of COVID-19 infection observed in people with chronic morbidities, through the waves and for both sexes, could also be related to a higher probability of testing in the profile of these patients. 

### 4.3. COVID-19 Inequalities

In terms of inequalities at the individual level, employees with low salaries presented the highest risk of COVID-19 confirmed diagnosis, especially in wave 2. The second wave in Aragón started with a series of outbreaks among seasonal workers. Seasonal agricultural workers in Spain are mainly migrants, with temporary, low-paid jobs with very poor health and hygienic conditions. These characteristics make them a particularly vulnerable group and, although special COVID protocols were implemented, they were clearly insufficient [29]. Likewise, employment status has been considered especially problematic in the COVID-19 pandemic, due to its relationship with class inequalities in income, employment conditions and safety [30]. The lockdown and the general recommendation of “working from home” has exacerbated the differences between those people who can do telematic work and those who cannot [31]. This is related to the fact that those low-paid workers are less likely to be in jobs where it is possible to work from home [32], with a higher risk of COVID-19 infection. Finally, those individuals belonging to lower socioeconomic groups are more likely to have unstable working conditions and income. In Spain, the effect of COVID-19 on employment rates has been huge. According to the Economically Active Population Survey [33], the number of workers in Spain decreased by more than 622,000 people during 2020. This financial uncertainty has been linked to worse mental health conditions and high stress levels, with a high likelihood of health risk behaviors [34]. Therefore, as some authors have pointed out [35], poverty not only increases exposure to the virus, but it also reduces immunity, which can be translated into a higher risk of COVID-19 infection. 

When inequalities in BHA of residence are evaluated, we observed that deprivation level of BHA influenced the risk of COVID-19 infection, showing statistical differences between the least deprived BHA and the most deprived. The association between deprived areas and high incidence rates of COVID-19 has already been described by other authors [17,18,20]. People living in deprived areas are more likely to live in precarious conditions, which involve overcrowded accommodation and limited access to outdoor space [28]. BHA inequities could be associated with differential exposure to the virus and differential susceptibility to infection [28]. Finally, BHA type (rural or urban) did not play a significant role in the risk of COVID-19 infection in Aragón. 

### 4.4. Strengths and Limitations

The main strength of this study lies in the fact that we analyzed all the individuals tested for COVID-19 in a population of 1.3 million people, including data from administrative health data sources and electronic health records. In addition, we used a combination of two different variables (information used to calculate pharmacy copayment levels and the type of user of the Aragón health service) to categorize the socioeconomic level of the individuals. This provides a better approximation to the real socioeconomic position of the individual.

Also, multilevel regression models allowed us to explore the impact of inequities on COVID-19 infection at different levels. Nonetheless, some aspects must be taken into consideration. First, two of the models presented (those corresponding to women for the overall period analyzed and for men at wave 3) were “singular”. Despite of this fact, we presented them in order to maintain comparability across models and waves, but their results must be interpreted cautiously. Second, the values of intraclass correlation coefficient (ICC) values obtained were low, but similar to those of other health studies. We have computed other measures, such as median odds ratio, which is considered an epidemiologically more suitable option for obtaining measures of variance in logistic regression, since it does not depend statistically on the prevalence of the outcome and allows the variance to be expressed at the area level on the well-known OR scale. Therefore, it permits comparison of the magnitude of area level variations with the impact of specific factors [36]. The median odds ratio quantifies the variation between clusters (the second-level variation) by comparing two people from two different clusters randomly chosen. In our study, the median odds ratio quantifies differences (i.e., variance $\sigma^{2}$) between deprivation quartile and zone of residence by comparing two individuals with the same covariates but from two different, randomly chosen deprivation quartiles or zones of residence. It is well known that individuals within a specific context may be more similar to each other than to individuals from a different context. Therefore, the interpretation of variance in multilevel analysis is pertinent to obtain information about a possible general effect of the context on individual outcomes [37].

It is also necessary to take into account some limitations inherent to observational studies, such as the quality of the data, which may have changed across the waves, or the existence of incomplete cases. Finally, as it has been previously stated, the cohort is integrated by all those individuals who were tested in Aragón. Although the age structure of the Aragón population is similar to the age distribution observed in the Aragón-COVID19, differences in other variables considered could exist between the reference population and the tested population. For this reason, different results could have been found if the entire population had been tested. 

## 5. Conclusions

Our study shows the existence of inequalities in the risk of COVID-19 confirmed infection, both at individual and area level. As Marmot et al. [32] have stated, the COVID-19 pandemic exposes and amplifies the existing inequalities in society. This requires the implementation of coordinated measures in the control, diagnosis and treatment of the epidemic, in order to avoid increasing inequalities, as well as the identification of vulnerable groups that will require more economic assistance to recover from the pandemic. In this sense, at the individual level, ensuring safe employment conditions and financial protection during pandemic is crucial [38,39]. Additionally, regarding measures at the area of residence, disease control efforts should be more intensive in those areas where the most vulnerable population lives [17] and adequate accessibility to diagnosis and treatment should be guaranteed. It will be necessary to monitor the measures implemented in order to know their impact on health inequalities in the short and medium term. Finally, we must not overlook the fact that a post-COVID scenario will probably lead to a new global economic crisis, especially if austerity measures are implemented again [14,40]. It is crucial, therefore, to learn from mistakes of the past and promote a change of scenario, where increasing social services for the whole population becomes a reality.

## Figures and Tables

**Table 1:** Sociodemographic and morbidity description of confirmed COVID-19 women. Differences by waves.

	Wave 1 (Number = 3,514)	Wave 2 (Number = 18,739)	Wave 3 (Number = 17,339)	*p*
Age (years old)				0.000
<15	16 (0.46%)	2321 (12.39%)	1752 (10.10%)	
15–44	850 (24.19%)	7738 (41.29%)	6252 (36.06%)	
45–64	1134 (32.27%)	4918 (26.24%)	5171 (29.82%)	
65–79	449 (12.78%)	1739 (9.28%)	1859 (10.72%)	
≥80	1065 (30.31%)	2023 (10.80%)	2305 (13.29%)	
Socioeconomic Level				<0.001
Employed ≥ €18,000 per year	716 (20.38%)	3237 (17.27%)	3666 (21.14%)	
Employed < €18,000 per year	931 (26.49%)	7872 (42.01%)	6406 (36.95%)	
Unemployed	70 (1.99%)	854 (4.56%)	685 (3.95%)	
Pensioner ≥ €18,000 per year	296 (8.42%)	932 (4.97%)	1047 (6.04%)	
Pensioner < €18,000 per year	1210 (34.43%)	3230 (17.24%)	3545 (20.45%)	
Mutualist	101 (2.87%)	299 (1.60%)	440 (2.54%)	
Free medicines	100 (2.85%)	1040 (5.55%)	665 (3.84%)	
Other	90 (2.56%)	1275 (6.80%)	885 (5.10%)	
Deprivation quartile				<0.001
Quartile 1 (least deprivation)	1149 (32.92%)	4324 (23.15%)	4792 (27.74%)	
Quartile 2	854 (24.47%)	4014 (21.49%)	4345 (25.15%)	
Quartile 3	608 (17.42%)	4051 (21.68%)	3417 (19.78%)	
Quartile 4 (highest deprivation)	879 (25.19%)	6293 (33.68%)	4723 (27.34%)	
Zone of residence				<0.001
Rural	886 (25.39%)	4226 (22.62%)	4875 (28.22%)	
Urban	2604 (74.61%)	14,456 (77.38%)	12,402 (71.78%)	
Weight complexity *	5.57 [2.50; 10.48]	3.47 [1.59; 6.61]	3.65 [1.61; 7.17]	<0.001
Presence of chronic morbidities	2977 (87.97%)	13,862 (78.72%)	9951 (80.50%)	<0.001
Presence of respiratory illnesses	402 (11.88%)	1580 (8.97%)	1185 (9.59%)	<0.001

*p*: statistical significance. Chi square and Mann–Whitney test; * Results expressed as median [interquartile range].

**Table 2:** Sociodemographic and morbidity description of confirmed COVID-19 men. Differences by waves.

	Wave 1 (Number = 2,473)	Wave 2 (Number = 16,846)	Wave 3 (Number = 15,128)	*p*
Age (years old)				0.000
<15	38 (1.54%)	2272 (13.49%)	1844 (12.19%)	
15–44	469 (18.96%)	7021 (41.68%)	5618 (37.14%)	
45–64	784 (31.70%)	4709 (27.95%)	4606 (30.45%)	
65–79	536 (21.67%)	1747 (10.37%)	1831 (12.10%)	
≥80	646 (26.12%)	1097 (6.51%)	1229 (8.12%)	
Socioeconomic Level				0.000
Employed ≥ €18,000 per year	494 (19.98%)	4518 (26.82%)	4737 (31.31%)	
Employed < €18,000 per year	366 (14.80%)	6370 (37.81%)	4794 (31.69%)	
Unemployed	46 (1.86%)	659 (3.91%)	457 (3.02%)	
Pensioner ≥ €18,000 per year	422 (17.06%)	1245 (7.39%)	1338 (8.84%)	
Pensioner < €18,000 per year	829 (33.52%)	2164 (12.85%)	2362 (15.61%)	
Mutualist	193 (7.80%)	381 (2.26%)	473 (3.13%)	
Free medicines	53 (2.14%)	587 (3.48%)	360 (2.38%)	
Other	70 (2.83%)	922 (5.47%)	607 (4.01%)	
Deprivation quartile				<0.001
Quartile 1 (least deprivation)	724 (30.08%)	3752 (22.42%)	4085 (27.25%)	
Quartile 2	609 (25.30%)	3512 (20.99%)	3807 (25.40%)	
Quartile 3	499 (20.73%)	3684 (22.01%)	3030 (20.21%)	
Quartile 4 (highest deprivation)	575 (23.89%)	5787 (34.58%)	4067 (27.13%)	
Zone of residence				<0.001
Rural	738 (30.66%)	4238 (25.32%)	4451 (29.70%)	
Urban	1669 (69.34%)	12,497 (74.68%)	10,538 (70.30%)	
Weight complexity *	5.56 [2.28; 10.74]	2.74 [1.11; 5.34]	2.88 [1.16; 5.99]	<0.001
Presence of chronic morbidities	1891 (85.53%)	11,192 (72.16%)	7755 (75.22%)	<0.001
Presence of respiratory illnesses	368 (16.64%)	1542 (9.94%)	1124 (10.90%)	<0.001

*p*: statistical significance; * Results expressed as median [interquartile range].

**Table 3:** Risk of having a COVID-19 confirmed diagnosis. Multilevel analyses in women for all the period analyzed and by wave.

Predictors	Global	Wave 1	Wave 2	Wave 3
	Odds Ratios (95% Confidence Interval)	Odds Ratios (95% Confidence Interval)	Odds Ratios (95% Confidence Interval)	Odds Ratios (95% Confidence Interval)
Intercept	0.16 *** (0.15–0.17)	0.01 *** (0.01–0.02)	0.13 *** (0.10–0.16)	0.30 *** (0.26–0.33)
Age (Ref: <15)				
15–44	1.51 *** (1.45–1.58)	9.47 *** (5.49–16.33)	1.63 *** (1.55–1.72)	1.47 *** (1.36–1.59)
45–64	1.61 *** (1.54–1.68)	12.94 *** (7.51–22.30)	1.67 *** (1.57–1.77)	1.68 *** (1.55–1.82)
65–79	1.57 *** (1.47–1.69)	16.26 *** (9.23–28.64)	1.55 *** (1.42–1.71)	1.64 *** (1.46–1.86)
≥80	2.50 *** (2.33–2.68)	32.93 *** (18.71–57.94)	2.26 *** (2.05–2.49)	3.00 *** (2.64–3.40)
Socioeconomic level (Ref: employed ≥ €18,000 per year)				
Employed < €18,000 per year	1.25 *** (1.21–1.30)	1.14 * (1.03–1.27)	1.31 *** (1.25–1.37)	1.13 *** (1.07–1.20)
Unemployed	1.23 *** (1.15–1.32)	0.91 (0.71–1.18)	1.29 *** (1.19–1.41)	1.10 (0.98–1.24)
Pensioner ≥ €18,000 per year	0.94 (0.88–1.01)	0.82 * (0.68–1.00)	0.95 (0.86–1.05)	0.89 * (0.79–1.00)
Pensioner < €18,000 per year	1.02 (0.97–1.09)	0.88 (0.74–1.05)	1.02 (0.94–1.10)	0.99 (0.90–1.10)
Mutualist	0.86 * (0.76–0.96)	0.92 (0.62–1.36)	0.71 *** (0.59–0.84)	0.88 (0.73–1.07)
Free medicines	1.27 *** (1.20–1.36)	0.87 (0.69–1.10)	1.39 *** (1.28–1.51)	1.20 ** (1.06–1.36)
Other	1.27 *** (1.20–1.35)	0.76 * (0.60–0.96)	1.39 *** (1.29–1.50)	1.16 ** (1.04–1.29)
Chronic morbidities	0.84 *** (0.82–0.87)	0.82 *** (0.73–0.92)	0.85 *** (0.82–0.89)	0.88 *** (0.83–0.94)
Random Effects				
τ_00_	0.0049 _BHA deprivation_	0.0033 _BHA deprivation_	0.0209 _BHA deprivation_	0.0025 _BHA deprivation_
	0.0000 _rural/urban BHA_	0.0043 _rural/urban BHA_	0.0123 _rural/urban BHA_	0.0021 _rural/urban BHA_
ρ (rho)	NA	0.0023	0.0100	0.0014
Number of observations	171,561	32,681	98,906	39,974
Marginal R^2^/Conditional R^2^	0.015/NA	0.107/0.109	0.017/0.027	0.018/0.020
MOR Deprivation quartile	1.0700	1.0562	1.1500	1.0484
MOR Zone of residence	1.0000	1.0642	1.1100	1.0445
LR test (Prob $>\chi^{2}$)	0.000	0.022	0.000	0.001

Ref: reference category; * *p* < 0.05; ** *p* < 0.01; *** *p* < 0.001; τ_00:_ between-group variance; ρ(rho): intraclass correlation coefficient (ICC); MOR: median odds ratios; LR test: likelihood ratio test; BHA: basic healthcare area; NA: not available.

**Table 4:** Risk of having a COVID-19 confirmed diagnosis. Multilevel analyses in men for all the period analyzed and by wave.

Predictors	Global	Wave 1	Wave 2	Wave 3
	Odds Ratios (95% Confidence Interval)	Odds Ratios (95% Confidence Interval)	Odds Ratios (95% Confidence Interval)	Odds Ratios (95% Confidence Interval)
Intercept	0.15 *** (0.14–0.17)	0.03 *** (0.02–0.04)	0.12 *** (0.10–0.15)	0.31 *** (0.28–0.33)
Age (Ref: <15)				
15–44	1.73 *** (1.66–1.81)	3.41 *** (2.40–4.85)	1.95 *** (1.85–2.06)	1.37 *** (1.27–1.48)
45–64	2.05 *** (1.96–2.14)	5.73 *** (4.05–8.10)	2.31 *** (2.18–2.45)	1.77 *** (1.63–1.92)
65–79	1.92 *** (1.79–2.06)	6.28 *** (4.30–9.17)	2.20 *** (1.99–2.43)	1.67 *** (1.47–1.89)
≥80	2.52 *** (2.34–2.72)	10.89 *** (7.44–15.94)	2.51 *** (2.25–2.80)	2.51 *** (2.19–2.88)
Socioeconomic level (Ref: employed ≥ €18,000 per year)				
Employed < €18,000 per year	1.12 *** (1.08–1.16)	0.87 (0.75–1.02)	1.18 *** (1.13–1.24)	1.05 (0.99–1.12)
Unemployed	1.03 (0.95–1.11)	0.89 (0.64–1.24)	1.10* (1.00–1.21)	0.93 (0.81–1.07)
Pensioner ≥ €18,000 per year	0.87 *** (0.81–0.93)	1.08 (0.89–1.32)	0.81 *** (0.73–0.89)	0.92 (0.82–1.03)
Pensioner < €18,000 per year	0.87 *** (0.82–0.93)	1.02 (0.85–1.23)	0.81 *** (0.74–0.88)	0.99 (0.89–1.10)
Mutualist	0.86* (0.75–0.98)	1.36 (0.95–1.94)	0.80* (0.66–0.96)	0.86 (0.68–1.08)
Free medicines	1.08 (1.00–1.17)	0.97 (0.71–1.33)	1.16** (1.05–1.28)	1.15 (0.98–1.35)
Other	1.10** (1.03–1.18)	1.01 (0.75–1.36)	1.16** (1.06–1.26)	1.11 (0.98–1.26)
Chronic morbidities	0.86 *** (0.83–0.88)	0.74 *** (0.64–0.84)	0.85 *** (0.82–0.89)	0.92** (0.87–0.98)
Random Effects				
τ_00_	0.0069 _BHA deprivation_	0.0036 _BHA deprivation_	0.0255 _BHA deprivation_	0.0009 _BHA deprivation_
	0.0004 _rural/urban BHA_	0.0071 _rural/urban BHA_	0.0092 _rural/urban BHA_	0.0000 _rural/urban BHA_
ICC	0.0022	0.0032	0.0104	NA
Number of observations	143,222	22,481	87,156	33,585
Marginal R^2^/Conditional R^2^	0.018/0.021	0.099/0.102	0.027/0.038	0.015/NA
MOR Deprivation quartile	1.0800	1.0590	1.1600	1.0300
MOR Zone of residence	1.0200	1.0836	1.1000	1.0000
LR test (Prob $>\chi^{2}$)	0.000	0.015	0.000	0.312

Ref: reference category; * *p*<0.05; ** *p*<0.01; ***p<0.001; τ_00:_ between-group variance; ρ (rho): intraclass correlation coefficient (ICC); MOR: median odds ratios; LR test: likelihood ratio test; BHA: basic healthcare area; NA: not available.

## Data Availability

Aragon-COVID19 data is available under request to IACS.

## Appendix A

**Table A1:** Variables used.

Variables	Description	Categories
Age	Age categorized	under 15 15–44 45–64 65–79 80 years or older
Socioeconomic Level	Calculated on the basis of pharmacy copayment levels and Social Security benefits received, according to the type of user of the Aragón health service. From the combination of these two variables, 8 categories mutually exclusive were obtained	—Employed ≥ 18 K: employed individuals earning 18,000€ per year or more. —Employed < 18 K: employed individuals earning less than 18,000€ per year. —Unemployed: individuals receiving the unemployment allowance. —Pensioner ≥ 18 K: individuals with a contributory pension €18,000 per year or more —Pensioner < 18 K: individuals with a contributory pension less of €18,000 per year —Mutualist: individuals affiliated to the mutual insurance system for civil servants. —Free medicines: individuals receiving free medicines (people with minimum integration income or who no longer receive the unemployment allowance) —Other: other situations not previously considered
Deprivation quartile	The deprivation index of the Basic Healthcare Area (BHA) of residence. This deprivation index combines information of four indicators from the Population and Housing Census 2011 (last available): % of unemployment, % of temporary workers, % of people between 16 and 64 years with low educational level and % of immigrants [18].	—Q1 (least deprived). —Q2 —Q3 —Q4 (most deprived).
Zone of residence	Classification of the zone of residence into rural or urban, according to the Aragon Government [19].	—Urban areas: areas are those that concentrate at least 80% of the BHA population in their municipalities. —Rural areas: areas are those that do not meet this criterion.
Weight complexity	Obtained from the morbidity adjusted groups (GMA) [17]. This source of information considers all medical diagnoses available from Primary Healthcare and hospital discharge records (CMBD). We considered GMA information from January 2020 in order to know the status prior to the COVID-19 diagnosis of the cohort individuals	Obtained from the aggregation of the patient´s different diagnoses
Chronic morbidities and		Presence of chronic morbidities (Yes/No) Presence of respiratory illnesses (Yes/No)
respiratory illnesses		
Hospitalization	This source of information considers hospital discharge records (CMBD).	Hospitalization (Yes/No)

**Table A2:** Sociodemographic and morbidity description of women tested for COVID-19 for the whole period analyzed.

	Global (Number = 193,184)	No COVID-19 (Number = 153,592)	COVID-19 Confirmed (Number = 39,592)	*p*
Age (years old)				0.001
<15	26,409 (13.67%)	22,320 (14.53%)	4089 (10.33%)	
15–44	70,202 (36.34%)	55,362 (36.04%)	14,840 (37.48%)	
45–64	53,308 (27.59%)	42,085 (27.40%)	11,223 (28.35%)	
65–79	22,002 (11.39%)	17,955 (11.69%)	4047 (10.22%)	
≥80	21,263 (11.01%)	15,870 (10.33%)	5393 (13.62%)	
Socioeconomic Level				<0.001
Employed ≥ 18 K	43,178 (22.35%)	35,559 (23.15%)	7619 (19.24%)	
Employed < 18 K	70,445 (36.47%)	55,236 (35.96%)	15,209 (38.41%)	
Unemployed	7499 (3.88%)	5890 (3.83%)	1609 (4.06%)	
Pensioner ≥ 18 K	12,211 (6.32%)	9936 (6.47%)	2275 (5.75%)	
Pensioner < 18 K	36,570 (18.93%)	28,585 (18.61%)	7985 (20.17%)	
Mutualist	4851 (2.51%)	4011 (2.61%)	840 (2.12%)	
Free medicines	8121 (4.20%)	6316 (4.11%)	1805 (4.56%)	
Other	10,309 (5.34%)	8059 (5.25%)	2250 (5.68%)	
Deprivation quartile				<0.001
Quartile 1 (least deprivation)	53,757 (27.97%)	43,492 (28.47%)	10,265 (26.02%)	
Quartile 2	46,764 (24.33%)	37,551 (24.58%)	9213 (23.35%)	
Quartile 3	38,623 (20.10%)	30,547 (20.00%)	8076 (20.47%)	
Quartile 4 (highest deprivation)	53,047 (27.60%)	41,152 (26.94%)	11,895 (30.15%)	
Zone of residence				0.779
Rural	48,548 (25.26%)	38,561 (25.25%)	9987 (25.32%)	
Urban	143,643 (74.74%)	114,181 (74.75%)	29,462 (74.68%)	
Weight complexity *	3.75 [1.72; 7.11]	3.76 [1.74; 7.09]	3.71 [1.65; 7.17]	0.126
Presence of chronic morbidities	137,836 (80.34%)	111,046 (80.35%)	26,790 (80.32%)	0.906
Presence of respiratory illnesses	18,193 (10.60%)	15,026 (10.87%)	3167 (9.49%)	<0.001
Hospitalization	4633 (2.40%)	223 (0.15%)	4410 (11.14%)	<0.001

*p*: statistical significance; * Results expressed as median [interquartile range].

**Table A3:** Sociodemographic and morbidity description of men tested for COVID-19 for the whole period analyzed.

	Global (Number = 164,805)	No COVID-19 (Number = 130,358)	COVID-19 Confirmed (Number = 34,447)	*p*
Age (years old)				<0.001
<15	29,159 (17.69%)	25,005 (19.18%)	4154 (12.06%)	
15–44	58,489 (35.49%)	45,381 (34.81%)	13,108 (38.05%)	
45–64	42,354 (25.70%)	32,255 (24.74%)	10,099 (29.32%)	
65–79	21,525 (13.06%)	17,411 (13.36%)	4114 (11.94%)	
≥80	13,278 (8.06%)	10,306 (7.91%)	2972 (8.63%)	
Socioeconomic Level				<0.001
Employed ≥ 18 K	47,983 (29.12%)	38,234 (29.33%)	9749 (28.30%)	
Employed < 18 K	52,053 (31.58%)	40,523 (31.09%)	11,530 (33.47%)	
Unemployed	5593 (3.39%)	4431 (3.40%)	1162 (3.37%)	
Pensioner ≥ 18 K	15,401 (9.34%)	12,396 (9.51%)	3005 (8.72%)	
Pensioner < 18 K	26,389 (16.01%)	21,034 (16.14%)	5355 (15.55%)	
Mutualist	5340 (3.24%)	4293 (3.29%)	1047 (3.04%)	
Free medicines	4884 (2.96%)	3884 (2.98%)	1000 (2.90%)	
Other	7162 (4.35%)	5563 (4.27%)	1599 (4.64%)	
Deprivation quartile				<0.001
Quartile 1 (least deprivation)	44,253 (27.14%)	35,692 (27.68%)	8561 (25.08%)	
Quartile 2	39,532 (24.24%)	31,604 (24.51%)	7928 (23.23%)	
Quartile 3	33,917 (20.80%)	26,704 (20.71%)	7213 (21.13%)	
Quartile 4 (highest deprivation)	45,371 (27.82%)	34,942 (27.10%)	10,429 (30.56%)	
Zone of residence				0.076
Rural	45,669 (28.01%)	36,242 (28.11%)	9427 (27.62%)	
Urban	117,404 (71.99%)	92,700 (71.89%)	24,704 (72.38%)	
Weight complexity*	3.08 [1.30; 6.19]	3.12 [1.32; 6.25]	2.92 [1.19; 5.96]	<0.001
Presence of chronic morbidities	107,536 (75.08%)	86,698 (75.26%)	20,838 (74.34%)	0.001
Presence of respiratory illnesses	17,008 (11.88%)	13,974 (12.13%)	3034 (10.82%)	<0.001
Hospitalization	5029 (3.05%)	135 (0.10%)	4894 (14.21%)	<0.001

*p*: statistical significance; * Results expressed as median [interquartile range].
